# Uncovering and Testing the Fuzzy Clusters Based on Lumped Markov Chain in Complex Network

**DOI:** 10.1371/journal.pone.0082964

**Published:** 2013-12-31

**Authors:** Fan Jing, Xie Jianbin, Wang Jinlong, Qu Jinshuai

**Affiliations:** 1 University Key Laboratory of Wireless Sensor Networks in Yunnan Province, Yunnan University of Nationalities, Kunming, P.R. China; 2 School of Urban Construction and Management, Yunnan University, Kunming, P.R. China; Semmelweis University, Hungary

## Abstract

Identifying clusters, namely groups of nodes with comparatively strong internal connectivity, is a fundamental task for deeply understanding the structure and function of a network. By means of a lumped Markov chain model of a random walker, we propose two novel ways of inferring the lumped markov transition matrix. Furthermore, some useful results are proposed based on the analysis of the properties of the lumped Markov process. To find the best partition of complex networks, a novel framework including two algorithms for network partition based on the optimal lumped Markovian dynamics is derived to solve this problem. The algorithms are constructed to minimize the objective function under this framework. It is demonstrated by the simulation experiments that our algorithms can efficiently determine the probabilities with which a node belongs to different clusters during the learning process and naturally supports the fuzzy partition. Moreover, they are successfully applied to real-world network, including the social interactions between members of a karate club.

## Introduction

The theory of network science has significantly improved our understanding of complex systems. In the last fifteen years, an explosive growth of interests and activities on the structure and dynamics of complex networks is appearing. One of the most favorable but challenging tasks in network science is cluster analysis, which is aimed at revealing possible partitions of a network into subsets of nodes (clusters, or communities) [1]. Markov chains are frequently used as analytic models in the quantitative evaluations of stochastic systems. Examples of their use may be found in diverse areas such as computer, biological, physical and social sciences as well as in business, economics and engineering [2]–[4]. The fundamental property characterizing the model, referred to as the Markov property, is that given the present, past and future transitions of the system are independent of each other [5], [6]. The information that is often sought from this model is either the transient or stationary probability of the system being in a given state. When the number of states is small, it is relatively easy to obtain the transient and stationary solutions allowing the prediction of the system behavior. However, as models become more complex the process of obtaining these solutions becomes much more difficult. There is also a wide class of situations, where the modeler does not need information about each state of the system but about classes of states only. This leads to the consideration of a new process, to be called the aggregated or lumped, whose states are the state classes of the original Markov chain. The new stochastic process need not to be Markovian. In order to be able to utilize all the power of the Markov chain theory, it is important to be able to claim that for a given initial distribution the aggregated process has the Markov property.

In a previous paper [7], a *k*-means approach is proposed to partition the networks based on optimal prediction theory proposed by Chorin and coworkers [8], [9]. The basic idea is to associate the network with the random walker Markovian dynamics [10], then introduce a metric on the space of Markov chains (stochastic matrices), and optimally reduce the chain under this metric. The final minimization problem is solved by an analogy to the traditional *k*-means algorithm [11], [12] in clustering analysis. This approach is motivated by the diffusion maps [13] and MNCut algorithms in imaging science [5].

To give a coarse definition about the study of complex networks from the viewpoints of applied mathematics, it is about the research of dynamical systems on graphs. The graph structure may be fixed, or time-varying; the dynamical system may be deterministic, or stochastic. Since these networks are typically very complex, it is of great interest to see whether they can be reduced to much simpler systems [5]–[7], [13]–[20]. And in a broader aspect, it is also closely related to the model reduction theory of differential equations [21], [22]. The concept of lumpability on hard partitions is a useful tool to analysis the dynamic of the network based on the a coarse grain view. Lumped Markov chain model of the random walker (i.e., a reduced-order Markov chain in which the clusters of the original network become nodes) which is easily derived from the original (highorder) Markov chain model. This notion of proximity of ‘nodes’ in the lumped markov chain reflects the intrinsic geometry of the meta-node set in terms of connectivity of the cluster in a diffusion process.

In the current paper, we first analyze the property of lumped markov chain and propose two novel ways of inferring the lumped markov transition matrix. Some useful results are proposed based on the analysis of the properties of the lumped markov process. Furthermore, we construct two algorithms — the steepest descent method with projection (SDP) and the reduced conjugate gradient method with projection (CGP) — from minimizing the objective function under the generalized framework in this paper. According to two choices of projection operators P1, P2, we obtain the formulations — SDP1, SDP2, CGP1, CGP2 — which have been applied to two artificial networks, including the ad hoc network, as well as real-world network, the karate club network. The proposed algorithms are easy to be implemented with reasonable computational effort and the final results do make sense in the considered models. It is demonstrated by these experiments that the algorithms can always perform successfully during the learning process and lead to a good clustering result.

## Materials and Methods

We will start with a brief review of markov random walks on complex network [8], [9]. Let 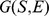 be a network with *N* nodes and *M* edges, where *S* is the nodes set, 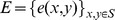 is the weight matrix and 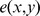 is the weight for the edge connecting the nodes *x* and *y*. A simple example of the weight matrix is given by the adjacency matrix: 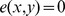 or 1, depending whether *x* and *y* are connected. We can relate this network to a discrete-time Markov chain with stochastic matrix *p* with entries 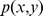 given by
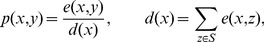
(1)where 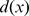 is the degree of the node *x* [10]–[13], [23]. This Markov chain has stationary distribution
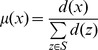
(2)and it satisfies the detailed balance condition
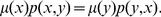
(3)


**Figure 1:**
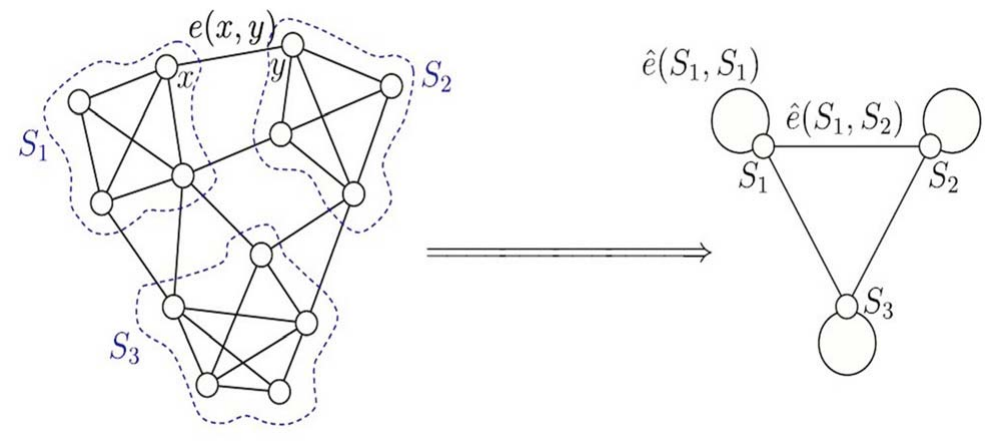
Lumped markov process of a network. For a given partition 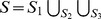 on a network 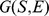, we define a lumped network 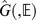 by aggregating all nodes belonging to a subset 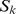 into a meta-node. The new weights 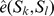 are computed via weight averaging the transition probabilities between nodes 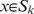 and 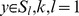
–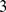, and the lumped Markov chain with transition probabilities 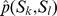 can also be obtained.

We take a partition of *S* as 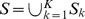 with 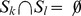 if 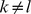. Our aim is to aggregate the nodes in each cluster in order to give the exact expression of lumped markov chain. To do so, first, we regard each set 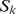 in the state space 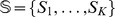 as corresponding to the nodes of a 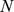-nodes network 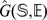, where 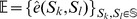, and the weight 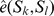 on the edge that connects 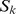 and 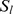 is defined as
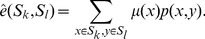
(4)where the sum involves all the transition probabilities between 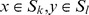. From the detailed balance condition (4), it can be verified that 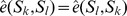. By setting 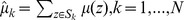, one can define the lumped Markov chain on graph 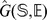 with stationary distribution 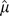 and lumped transition probabilities
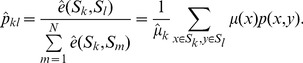
(5)It can be easily shown that 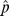 is a stochastic matrix on the state space 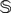 and satisfies a detailed balance condition with respect to 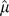, i.e.
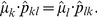
(6)This construction of a lumped markov chain is shown in Figure 1.

To give a more clear probability expression, we denote such probability function as 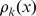 to represent the probability which the node *x* belongs to the *k*-th cluster with. Naturally we need the assumption that
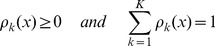
(7)for all 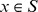.

Thus, the lumped markov transition matrix can be rewritten based on 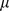


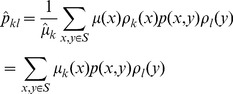
(8)


Another approach of deriving the lumped markov transition matrix is based on lifted transition matrix. Any lumped stochastic matrix 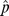 can be naturally lifted to the space of stochastic matrices on the original state space *S* via
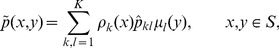
(9)where
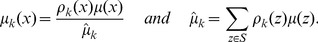
(10)


The basic idea in [24], [25] is to introduce a metric, also called the Hilbert-Schmidt norm, in the space of stochastic matrices. The optimal partition and the corresponding reduced Markov chain 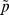 is found by minimizing
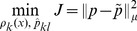
(11)where
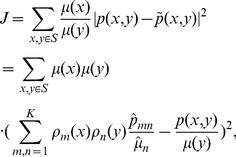
(12)subject to the constraint Eq.(7).

To minimize the objective function *J* in Eq.(12), we define
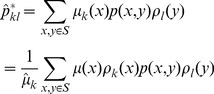
(13)which has the similar form as 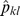 in hard clustering. Then 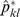 is indeed a stochastic matrix because 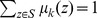 for all *k*. Furthermore we have that 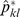 satisfies the detailed balance condition with respect to 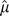, that is
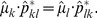
(14)With the above background, we have the following basic results.

The expressions of 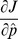 and 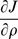 according to Eq.(12) are given by
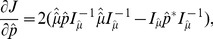
(15a)

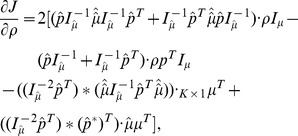
(15b)where 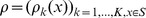 is a 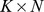 matrix and 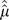 is a 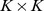 matrix with entries
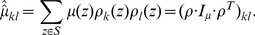
(17)The diagonal matrices 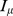, 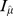 are 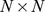 and 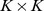 respectively, with entries
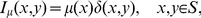
(17a)

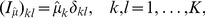
(17b)where 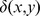 and 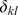 are both Kronecker delta symbols.

The proof of Lemma 1 can be found in Appendix_S1.pdf. Eq.(15a) and Eq.(15b) give the partial derivatives of the objective function in Eq.(12), which are the critical points of constructing gradient methods.

Based upon this formulation, we can find the optimal 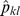 for any fixed partition 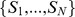. From the optimality condition
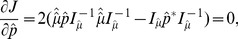
(18)we can obtain Eq.(8). It indicates that when the partition 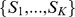 is known, the minimizer of Eq.(11) is unique, which can be given by Eq.(8), and the corresponding 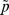 is the stochastic matrix in the class Eq.(9) which provides the best rank-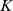 approximation of the original one under the metric Eq.(11).

### SDP

In this section, based on the lumped markov transition matrix 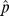 and corresponding results derived before, we devote to find ways to uncover partitions. An obvious choice to solve the constrained optimization Eq.(11) is the steepest descent method [12]. However, the components of 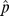 and 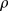 may become negative and non-normalized during the descent procedure for our problems, which makes the probabilistic interpretation useless. To ensure the nonnegativity and normalization conditions for 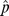 and 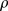, we add a projection step after each renewal. This leads to the following Steepest Descent method with Projections(SDP).

1. Set up the initial state 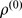 as the indicator matrix for each node in the network with the 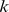-means algorithm in [7], 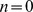.
2. Perform the following iteration until 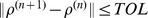:
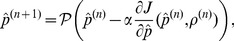
(19a)

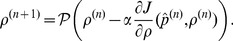
(19b)Here 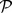 is some type of projection operator which maps a real vector into a probability vector, 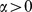 is the learning rate and 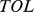 is a prescribed tolerance.
3. The final 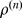 gives the fuzzy partition for each node.

Two choices of the projection operator 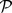 are used in our computations, but the final results seem to be insensitive to them. Now suppose 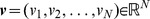, and 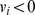 when 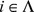, we make projection as any of the following two choices

P1 Direct projection to the boundary.

When 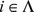, we set 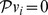; otherwise we set 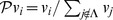.

P2 Iterative projections.

At first make projection to the hyperplane 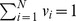, then check each component of the projected 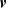. If 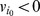, we take 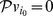 and make projection again to a dimension reduced hyperplane 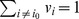. Repeat the projection procedure to a lower and lower dimensional hyperplane until no component is negative.

The application of projection operators for SD can simply solve the original constrained optimization Eq.(11), while to solve it exactly may involve much more complexity. Note that with this SDP method, we can not guarantee that 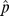 and 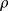 are the exact minimizer of the original problem Eq.(11) with non-negative and normalization constraints, but we take them as an approximate minimizer. The numerical results show that this strategy works fine for many examples.

The learning rate 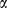 was usually chosen that started from a reasonable initial value and then reduced to zero with the iteration number 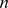 in such a way that 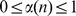, and
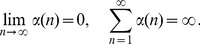
(20)The typical example of such case is 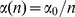, where 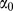 is a positive constant [26]. Another choice is to fix the learning rate 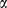 as a positive constant [24], [25], which we utilize here, since the initial partition is good enough that the objective function Eq.(12) descends much more slowly when the learning rate becomes smaller, while larger values of 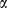 cause blow up.

The number of the iteration steps is difficult to be estimated, which may depend on the structure of the network itself, the choice of the initial values, etc. It usually converges fast for well-clustered networks and may converge slowly for diffusive networks.

Now let us estimate the computational cost in each iteration. In the iteration step for 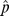, all of the matrices are of order 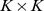 and full according to Eq.(15a). It is easy to find that the computation of 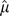 costs 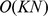, and the computation of 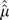 costs 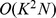. Note that the stochastic matrix 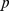 is sparse with 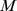 entries, so the computation for 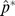 costs 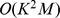, where 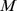 represents the number of edges, which is usually assumed 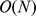 in realistic networks. So finally, we obtain the cost in the step for 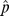 is 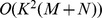. The cost for 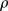 is also 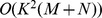 according to Eq.(15b), since 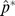 is involved in the equations.

### CGP

Another choice is to minimize the objective function using a simplified formulation of traditional conjugate gradient method, which is frequently used in machine learning [27]. It can be also regard as the above steepest descent method with a non-zero momentum term, which leads to the following reduced Conjugate Gradient method with Projections(CGP).

1. Set up the initial state 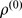 as the indicator matrix for each node in the network with the 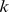-means algorithm in [13], 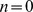.
2. Perform the following iteration until 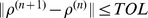:
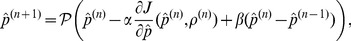
(21a)

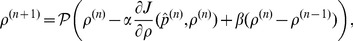
(21b)Here 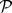 is some type of projection operator which maps a real vector into a probability vector, 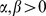 is the learning rates and 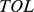 is a prescribed tolerance.
3. The final 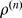 gives the fuzzy partition for each node.

We again note that this is just a reduced form of conjugate gradient method, and it is demonstrated by simulation experiments that such method performs more efficiently than SD, just like the superiority traditional conjugate gradient has. The learning rate 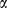 and 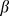 are still chosen as constants by experience due to the same reason mentioned above. The computational cost in each iteration of CGP is the same as SDP, which is also 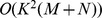 for both 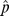 and 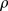. Associating the two projections described above with SDP and CGP respectively, we refer to the derived algorithms: SDP1, SDP2, CGP1, CGP2, as the fuzzy partitioning algorithms for networks.

## Results

In this section, simulation experiments on artificial network, the ad hoc network with 128 nodes, is carried out to demonstrate the performance of the proposed algorithms, via comparing the clustering results with some priori quantities. Moreover, the algorithms is applied to real-world network, the social interactions between members of a karate club.

### Ad hoc network with 128 nodes

The first example is the ad hoc network with 128 nodes. The ad hoc network is a benchmark problem used in many papers [7], [16], [17], [22]. It has a known partition and is constructed as follows. Suppose we choose 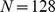 nodes, split them into 4 clusters with 32 nodes each. Assume that pairs of nodes belonging to the same clusters are linked with probability 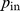, and pairs belonging to different clusters with probability 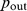. These values are chosen so that the average node degree 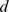 is fixed at 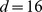. In other words, 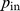 and 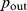 are related as
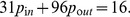
(22)We will denote 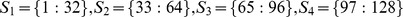.

**Figure 2:**
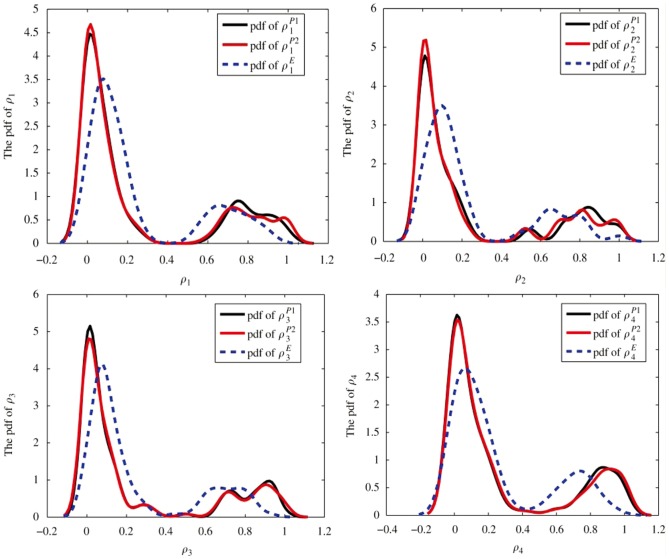
The pdf of 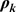 and 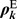 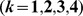 for the given ad hoc network with 128 nodes and 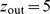. The solid lines and dashed lines represent the pdf of 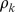 and 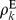 respectively. In each figure, the lower peak corresponds to the nodes in this cluster, and the higher peak corresponds to the other nodes outside of this cluster.

To test on a less diffusive network, we take 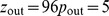 and generate the network according to Eq.(1). This network has a fuzzy clustering structure that some nodes should have immediate weights belonging to different clusters. We set the parameters by 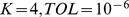, and the learning rates 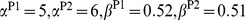 in this model computation. 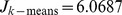 is obtained after initialization [12]. The numerical results are shown in Table 1. Here we compare 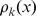 with an interesting quantity, the degree fraction 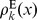, which is defined as
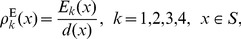
(23)where 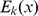 is the number of nodes that are connected with 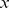 and lie in cluster 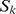. Thus we have 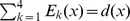. With this definition, 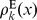 means the fraction of the edges connected with the node 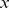 in the 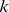-th cluster. Note that this is not the same as the clustering probability, even though it is an interesting quantity to be compared with. We expect that the degree fraction Eq.(2) is close to our result 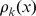 for network though generally this assertion needs to be justified or disconfirmed theoretically. To verify this fact, we define the mean and maximal 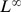-error of 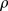


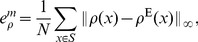
(24a)

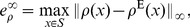
(24b)for error comparing. Table 1 shows that the deviation between these two is about 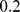. Obviously CG algorithm improves the convergence rate of SD. The projection P1 has the smallest 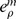, while the projection P2 reaches a better minimum which indicates a more accurate result. In Figure 2 we plot the probability distribution function (pdf) of 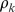 and 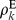 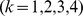. We observe that the shape of the pdf for 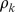 or 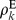 are almost the same. Note that all the 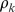
's have a lower peak centered at about 0.85, which corresponds to the nodes in this cluster, and a higher peak centered at about 0.05, which corresponds to the other nodes outside of this cluster. The case for 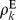 is similar but with the lower peak centered at about 0.7 and the higher peak centered at about 0.1. We note here that the center 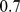 corresponds to the choice of the parameters 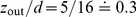. If we classify the nodes according to the majority rule, i.e. if 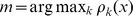 for a given node 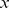 then we set 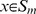, we obtain the 4-cluster partition exactly for this model. This also verifies the accuracy of our algorithms, but fuzzy algorithms give more detailed information for each node.

**Table 1:** 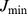
 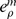 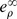.

	Iterations	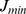	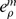	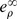
SDP1&CGP1	110&55	5.8794	0.1191	0.2389
SDP2&CGP2	119&54	5.8757	0.1207	0.2374

The iterations, the value of the objective function 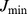 and the mean and maximum 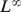-error of 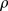 defined in Eq.(3) for algorithms.

### Karate club network

This network was constructed by Wayne Zachary after he observed social interactions between members of a karate club at an American university [28]. Soon after, a dispute arose between the clubs administrator and main teacher and the club split into two smaller clubs. It has been used in several papers to test the algorithms for finding clusters in networks [7], [14]–[18].

There are 34 nodes in karate club network, where each node represents one member in the club. In Zachary's original partition, each node belongs to only one sub-club after splitting. We label it as red or yellow color in the figures to show its attribute in the graph representation. From the viewpoint of the fuzzy clustering, the attribute of each node is no longer an indicator function but rather a discrete probability distribution. In our following notations, the association probability 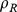 and 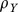 means the probability of each node belonging to red or yellow colored cluster respectively.

**Figure 3:**
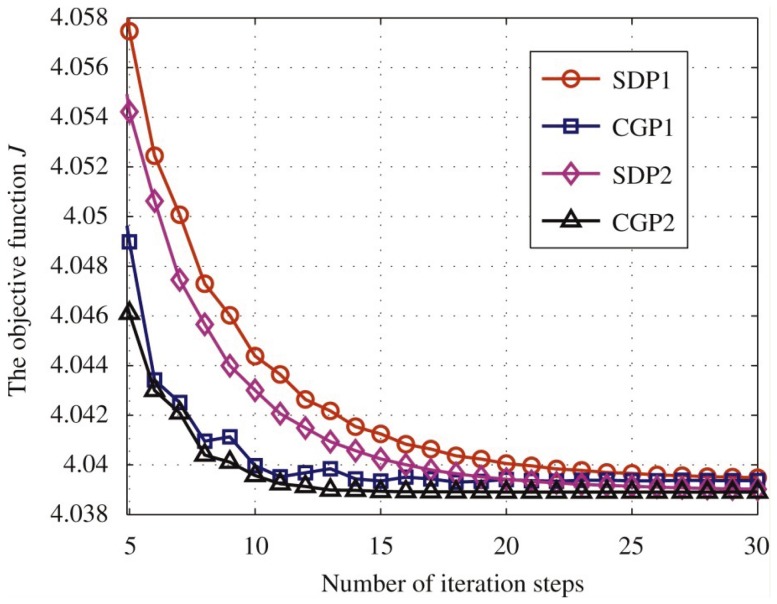
The convergence history of the objective function 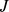 during 5–30 iterations of the karate club network.

**Figure 4:**
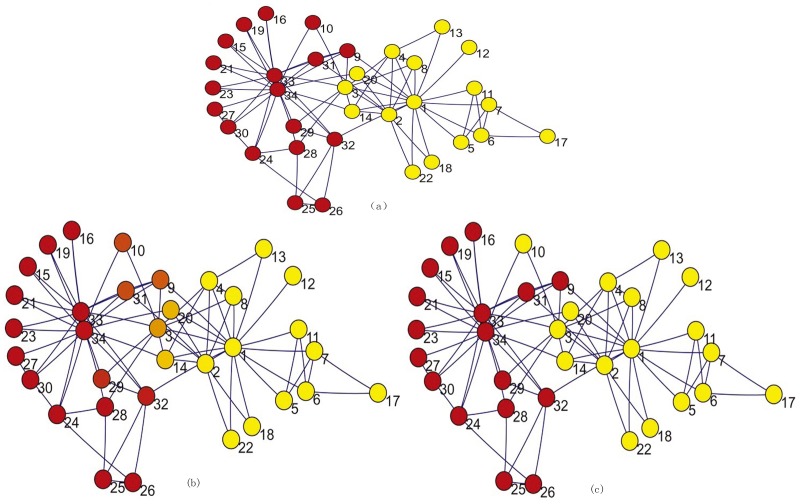
The visualization of the partitioning results of the karete club network. (a)Hard partition with thresholding operation according to the node's maximal weight. (b)Hard partition by *k*-means in [13]; (c)Fuzzy partition by CGP2.

We set the parameters by 
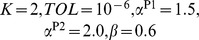
. Here 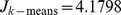 is obtained after initialization [7]. The numerical results are shown in Table 2. It can be demonstrate again that CGP is more efficient, and P2 can reach a smaller value of 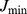. Figure 3 shows the convergence history during 5–30 iterations for the methods. It can be obviously seen that CGP, which decreased the objective function faster than SDP, performs more efficiently. The final association probabilities are presented in Table 3, where 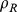 and 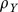 are the probability of belonging to the red or yellow colored group shown in Figure 4 respectively. Comparing 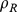 or 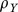 between P1 and P2, we find that almost all the errors are less than 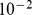, but the association probability 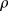 is quite different from the 0–1 distributions obtained in the 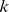-means algorithm. Now let us compare the association probability 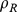 and 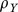 obtained by our methods with the original partition result obtained by Zachary. In [28], Zachary gave the partition 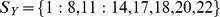 and 
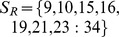
. If we classify the nodes according to the majority rule, i.e., if 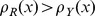 then we set 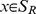, otherwise we set 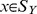, we obtain the same partition as Zachary's (see Figure 4(a)). We note that this hard partition deduced by fuzzy partition is more reasonable than the result of *k*-means(see Figure 4(b)), since node 10 is classified correctly this time.

**Table 2:** 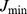
 of the karate club.

	Iterations	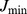
SDP1&CGP1	153&55	4.0394
SDP2&CGP2	173&53	4.0389

The iterations and minimized objective function values 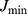 for the algorithms of the karate club network.

**Table 3:** The association probability.

	Nodes	1	2	3	4	5	6	7	8	9	10	11	12
SDP1&CGP1	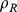	0.039	0.079	0.44	0	0	0	0	0.004	0.675	0.764	0	0
	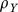	0.96	0.92	0.56	1	1	1	1	0.996	0.3254	0.236	1	1
SDP2&CGP2	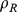	0.052	0.098	0.452	0.018	0	0	0	0.03	0.678	0.766	0	0
	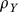	0.948	0.902	0.548	0.982	1	1	1	0.97	0.3222	0.234	1	1

The association probability that each node belongs to different clusters. 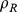 and 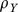 means the probability belonging to red or yellow colored cluster in Figure 4 respectively.

However, we have more detailed information in fact. From Table 3, we find 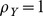 for nodes 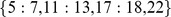, which lie at the boundary of the yellow colored group; and 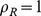 for nodes 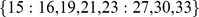, which mostly lie at the boundary of the red colored group. The others belong to the red and yellow colored groups with nonzero probability, especially the nodes 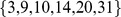 have more diffusive probability and they play the role of transition nodes between the red and yellow colored groups. We can visualize the data 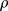 more transparently with the color vector Eq.(23) for each node. We can conclude from Figure 4(c) that how much probability each of the 34 members stands by both parts with. Members 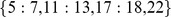 and 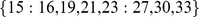 have an obvious attitude on following their leader, i.e. the club administrator or the main teacher. Others such as 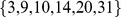 hold neutralism that they can support either leader according their weights.

## Discussion

We address the expression of lumped markov transition matrix for networks with two novel method in this paper. This can also be considered as a generalization of markov random walk dynamic in statistics for the networks. We successfully constructed the steepest descent method with projection (SDP) and the reduced conjugate gradient method with projection (CGP). They are derived to search for the local minimum of the objective function in Eq.(12) under the fuzzy clustering framework, which is extended from a deterministic framework for network partition based on the optimal prediction of a random walker Markovian dynamics [7]. The simulation experiments have shown that the algorithms can efficiently determine the fuzzy partition matrix. Partitioning the network with thresholding operation according to the node's maximal weight can give a more reasonable clustering result than the previous *k*-means algorithm [7]. We use two datasets(Ad hoc network with 128 nodes and Karate club network) to validate algorithms and achieve good results Numerical results show that our algorithms with two different projections produce similar results, while the CGP2 algorithm has better efficiency and accuracy. Moreover, the algorithms succeed in real-world learning tasks.

## Supporting Information

Text S1 **The ad hoc network is a benchmark problem used in many papers** [**8**] **,** [**9**] **,** [**12**] **,** [**16**] **.** It has a known partition and is constructed as follows. Suppose we choose *N* = 128 nodes, split them into 4 clusters with 32 nodes each.
(TXT)

Text S2 **The file karate.txt contains the network of friendships between the 34 members of a karate club at a US university, as described by Wayne Zachary in 1977.** If you use these data in your work, please cite W. W. Zachary, An information flow model for conflict and fission in small groups, Journal of Anthropological Research 33, 452–473 (1977).
(TXT)

Appendix S1 **The Proof of Lemma 1.**
(PDF)
