# Correction: Uncovering and Testing the Fuzzy Clusters Based on Lumped Markov Chain in Complex Network

**DOI:** 10.1371/journal.pone.0094154

**Published:** 2014-04-03

**Authors:** The PLOS ONE Staff

There are errors in the Funding section. The correct funding information is as follows: This work was supported by the National Natural Science Foundation of China (grant No.60963026, No.61163061, No.51264037), and the Application Basic Research Fund of Yunnan Province (grant No.2011FZ174). The National Natural Science Foundation of China: http://www.nsfc.gov.cn/. The funders had no role in study design, data collection and analysis, decision to publish, or preparation of the manuscript.
