# Supplementary material for: Uncovering and Testing the Fuzzy Clusters Based on Lumped Markov Chain in Complex Network
Source: PLoS One. 2013 Dec 31;8(12):e82964. doi: 10.1371/journal.pone.0082964 (PMC3877001; doi:10.1371/journal.pone.0082964)
Supplement: Appendix S1 — The Proof of Lemma 1. (PDF) [file pone.0082964.s003.pdf]

Appendix: Proof of Lemma 1

Fan Jing[1,*], Xie Jianbin[2], Wang Jinlong[1], Qu Jinshuai[1]
1 University key laboratory of wireless sensor networks in Yunnan province, Yunnan university of nationalities, Kunming 650031,Yunnan, P.R. China
2 School of urban construction and management, Yunnan university, Kunming, P.R .China,, Kunming 650091,Yunnan, P.R.China
∗ E-mail: Corresponding fanjing9476@163.com

Firstly we take the variation of J in Eq.(12) with respect to $\hat{p}_{kl}$

$$\begin{aligned}
&\frac{\partial J}{\partial \hat{p}_{kl}} \\
=\;& 2\sum_{x,y\in S}\mu(x)\mu(y)\Big(\sum_{m,n=1}^{K}\rho_m(x)\rho_n(y)\frac{\hat{p}_{mn}}{\hat{\mu}_n}-\frac{p(x,y)}{\mu(y)}\Big) \\
&\cdot\sum_{s,t=1}^{K}\rho_s(x)\rho_t(y)\frac{1}{\hat{\mu}_t}\delta_{sk}\delta_{tl} \\
=\;& 2\Big(\frac{1}{\hat{\mu}_l}\sum_{x,y\in S}\sum_{m,n=1}^{K}\mu(x)\mu(y)\rho_k(x)\rho_l(y)\rho_m(x)\rho_n(y)\frac{\hat{p}_{mn}}{\hat{\mu}_n} \\
&-\frac{1}{\hat{\mu}_l}\sum_{x,y\in S}\mu(x)p(x,y)\rho_k(x)\rho_l(y)\Big) \\
=\;& 2\Big(\frac{1}{\hat{\mu}_l}\sum_{m,n=1}^{K}\hat{\hat{\mu}}_{km}\frac{\hat{p}_{mn}}{\hat{\mu}_n}\hat{\hat{\mu}}_{nl}-\frac{\hat{\mu}_k}{\hat{\mu}_l}\hat{p}^*_{kl}\Big) \qquad (1)
\end{aligned}$$

Representing the above result with matrix form gives Eq.(15a).

Now we take the variation of J with respect to $\rho_r(z)$, which gives

$$\begin{aligned}
&\frac{\partial J}{\partial \rho_r(z)} \\
=\;& 2\sum_{x,y\in S}\mu(x)\mu(y)\Big(\sum_{m,n=1}^{K}\rho_m(x)\rho_n(y)\frac{\hat{p}_{mn}}{\hat{\mu}_n}-\frac{p(x,y)}{\mu(y)}\Big) \\
&\cdot\sum_{k,l=1}^{K}\Big[\delta_{kr}\delta(x,z)\rho_l(y)\frac{\hat{p}_{kl}}{\hat{\mu}_l}+\delta_{lr}\delta(y,z)\rho_k(x)\frac{\hat{p}_{kl}}{\hat{\mu}_l} \\
&-\rho_k(x)\rho_l(y)\frac{\hat{p}_{kl}}{\hat{\mu}_l^2}\sum_{w\in S}\delta_{lr}\delta(w,z)\mu(w)\Big], \qquad (2)
\end{aligned}$$

With the detail balance condition in Eq.(3) and the definition of $\hat{p}^*$ in Eq.(13), we actually have

$$
\begin{aligned}
&\frac{\partial J}{\partial \rho_r(z)} \\
=\;& 2\Bigg[\sum_{y\in S}\mu(z)\mu(y)\Bigg(\sum_{m,n=1}^{K}\rho_m(z)\rho_n(y)\frac{\hat{p}_{mn}}{\hat{\mu}_n}-\frac{p(z,y)}{\mu(y)}\Bigg) \\
&\cdot\sum_{k=1}^{K}\rho_l(y)\frac{\hat{p}_{rl}}{\hat{\mu}_l} \\
&+\sum_{x\in S}\mu(z)\mu(x)\Bigg(\sum_{m,n=1}^{K}\rho_m(x)\rho_n(z)\frac{\hat{p}_{mn}}{\hat{\mu}_n}-\frac{p(x,z)}{\mu(z)}\Bigg) \\
&\cdot\sum_{k=1}^{K}\rho_k(x)\frac{\hat{p}_{kr}}{\hat{\mu}_r} \\
&-\sum_{x,y\in S}\mu(x)\mu(y)\Bigg(\sum_{m,n=1}^{K}\rho_m(x)\rho_n(y)\frac{\hat{p}_{mn}}{\hat{\mu}_n}-\frac{p(x,y)}{\mu(y)}\Bigg) \\
&\cdot\sum_{k=1}^{K}\mu(z)\rho_k(x)\rho_r(y)\frac{\hat{p}_{kr}}{\hat{\mu}_r^2}\Bigg] \\
=\;& 2\Bigg[\sum_{l,m,n=1}^{K}\hat{\hat{\mu}}_{ln}\rho_l(y)\frac{\hat{p}_{mn}}{\mu_n}\frac{\hat{p}_{rl}}{\hat{\mu}_l}-\sum_{y\in S}\sum_{l=1}^{K}p(z,y)\rho_l(y)\frac{\hat{p}_{rl}}{\hat{\mu}_l} \\
&+\sum_{k,m,n=1}^{K}\hat{\hat{\mu}}_{km}\rho_k(x)\frac{\hat{p}_{mn}}{\hat{\mu}_n}\frac{\hat{p}_{kr}}{\hat{\mu}_r}-\sum_{x\in S}\sum_{k=1}^{K}p(z,x)\rho_k(x)\frac{\hat{p}_{kr}}{\hat{\mu}_r} \\
&-\sum_{k,m,n=1}^{K}\hat{\hat{\mu}}_{mk}\hat{\hat{\mu}}_{rn}\frac{\hat{p}_{mn}}{\hat{\mu}_n}\frac{\hat{p}_{kr}}{\hat{\mu}_r^2}+\sum_{k=1}^{K}\mu_k\hat{p}^*_{kr}\frac{\hat{p}_{kr}}{\hat{\mu}_r^2}\Bigg]\mu(z)
\end{aligned}
\tag{3}
$$

After suitable manipulations we obtain Eq.(15b) finally.
